# Research highlight: cultivating community-based participatory research (CBPR) to respond to the COVID-19 pandemic: an illustrative example of partnership and topic prioritization in the food services industry

**DOI:** 10.1186/s12889-024-17898-z

**Published:** 2024-03-18

Food service workers are pivotal in society, ensuring food access in numerous venues like grocery stores and restaurants. Nonetheless, during the COVID-19 pandemic, these workers had higher viral contact, resulting in increased infection and mortality rates in this group. This was notably the case in New Orleans, a city largely dependent on tourism and dining.

The authors established a collaboration with food service workers and their allies to better comprehend their experiences and concerns during the pandemic. The study was conducted from August 2021 to February 2023 using varied techniques—surveys, focus groups, and qualitative analysis—to pinpoint the key pandemic-related challenges these workers encountered.

The survey targeted individuals presently employed in New Orleans’ food service sector, with data gathered from February to April 2022, during the final phase of the Omicron BA.1 surge. Focus groups were conducted from April to October 2022, examining three primary issues: COVID-19 health and safety measures, stress and mental health during the pandemic, and the pandemic’s lasting impacts. Finally, a rapid qualitative assessment was conducted from December 2022 to January 2023, focusing on the problem of reducing in-home spread of COVID-19 when a household member tests positive.

The findings revealed that food service workers confronted significant challenges related to health and safety, stress and mental health, and the long-term effects of the pandemic. Employers frequently offered low-level droplet mitigation (measures to reduce spread of virus through droplets) instead of high-level airborne mitigation (measures to reduce spread of virus through air), creating high-risk exposure environments. These workers also had to make tough health and safety choices with limited clear public health guidance and structural supports. Moreover, they faced substantial stressors and mental health issues, with scarce counseling aid. Long-term effects of the pandemic were also noted in terms of mental health, Long COVID, and financial stress. Participants expressed a need for more support to prevent in-home COVID-19 spread and to receive more health, mental health, and financial aid in the food service sector.

This research is a successful example of an academic-community partnership during a crisis and underscores the significance of such collaborations in addressing urgent issues faced by vulnerable communities during public health crises. It guides future research, programs, and policies aimed at lessening the enduring impact of the COVID-19 pandemic and other airborne respiratory diseases among high-risk occupational exposure individuals.

*The following summaries of hand-selected papers were generated by Springer Nature's artificial intelligence tool and revised by a subject matter expert to meet Springer Nature’s standards.*

